# Recyclable Organic Redox Molecules for Sustainable Batteries

**DOI:** 10.1002/cssc.202402707

**Published:** 2025-03-31

**Authors:** Kouki Oka, Hitoshi Kasai

**Affiliations:** ^1^ Institute of Multidisciplinary Research for Advanced Materials Tohoku University 2-1-1 Katahira, Aoba-ku Sendai 980-8577 Miyagi Japan; ^2^ Carbon Recycling Energy Research Center Ibaraki University 4-12-1 Nakanarusawa Hitachi 316-8511 Ibaraki Japan; ^3^ Deuterium Science Research Unit Center for the Promotion of Interdisciplinary Education and Research Kyoto University Yoshida Kyoto 606-8501 Sakyo-ku Japan

**Keywords:** Organic redox molecule, Organic battery, Sustainable chemistry

## Abstract

Sustainable and environment‐friendly rechargeable devices are required to meet increasing electrical energy demands. Organic batteries are fabricated using organic redox materials which are potentially synthesised from earth‐abundant components. However, to avoid plastic pollution, these organic materials must display sufficient functions and ensure safe management post‐utilisation. This study demonstrated a sustainable and environment‐friendly recycling method for functional organic materials developed using organic redox molecules. These molecules could be prepared from earth‐abundant and sustainable raw chemicals via environment‐friendly preparation routes. The eco‐friendly battery, which uses organic redox molecules as anode‐ and cathode‐active materials and neutral aqueous solution as an electrolyte, exhibited high cyclability (>95 % capacity after 100 cycles) and high‐rate capability (15 C). After use, the electrode‐active material was separated and/or decomposed to the original raw chemicals, demonstrating a green and sustainable option to use conventional organic redox materials.

## Graphical abstract

This study demonstrated an environment‐friendly recycling method for organic materials developed using organic redox molecules. Organic redox molecules could be prepared from earth‐abundant raw chemicals via preparation routes with low environmental burden. The eco‐friendly and all‐organic battery fabricated using them exhibited high cyclability and rate capability. After use, they were separated and/or decomposed to the original raw chemicals.

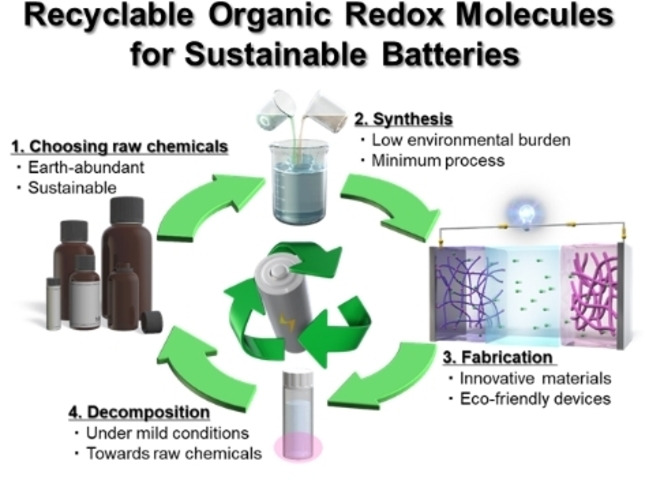

## Introduction

To meet the increasing global demands for electrical energy, rechargeable batteries are required. The rechargeable battery market is dominated by lead‐acid and lithium‐ion batteries.^[1]^ However, lead, which is the electrode‐active material in lead‐acid batteries, is highly toxic and pollutes the environment.^[2]^ The electrode‐active material in lithium‐ion batteries uses exhaustive resources, such as cobalt; moreover, the electrolyte used (organic solvent) is highly flammable.^[3]^ Furthermore, the technology to recycle electrode‐active materials used in lithium‐ion batteries is still under development, with the recycling rate of lithium being only 9 %, that of cobalt and nickel being around 20 %.[^4^, ^5^, ^6^] Considering the above‐mentioned concerns, next‐generation batteries should exhibit low toxicity, non‐flammability, high environmental compatibility, high utilisation of sustainable and earth‐abundant resources, and/or high recyclability to ensure long term sustainability.[^7^, ^8^, ^9^, ^10^]

Organic batteries, fabricated by organic redox materials as both anode‐ and cathode‐active materials, are a promising alternative and have been aggressively investigated and developed.[^8^, ^11^, ^12^, ^13^] Particularly, all‐organic batteries using neutral aqueous solution as an electrolyte are safe and environment‐friendly.[^14^, ^15^, ^16^, ^17^, ^18^, ^19^] (Available all‐organic aqueous batteries are listed in Table S1.) Organic redox materials comprise earth‐abundant building blocks (e.g., H, C, N, O, and S), and their electrochemical properties can be easily adjusted by changing their constituent atoms and molecular structures.[^9^, ^20^, ^21^, ^22^] Furthermore, the maturation technology for producing organic molecules from biomass[^23^, ^24^, ^25^] promotes sustainable production of organic redox materials. Polymers with organic redox molecules can easily fix these molecules at a high density in the fabricated electrode; therefore, organic redox polymers have gained increasing attention in the field of application of organic redox materials.[^8^, ^9^, ^13^]

However, owing to increasing plastic pollution, these functional organic materials should be designed not only to exhibit sufficient functions but also ensure safe management post‐utilisation.[^26^, ^27^, ^28^, ^29^] That is, the design strategy of functional organic materials and their devices should facilitate the decomposition of the materials into their raw chemical‐form post‐utilisation. In particular, sustainable and environment‐friendly organic electrode‐active materials are required, which can be prepared by environment‐friendly synthesis routes using sustainable and/or earth‐abundant raw chemicals, which can be decomposed to their original raw components after use through a facile method [e.g., heating at <300 °C, substantially lower than the high‐temperature heat treatment (>500 °C) for lithium‐ion battery waste.[^4^, ^5^] In this context, we focused on organic redox molecules, because their relatively simple structure, compared with that of their polymers, facilitated the design of a strategy for their facile and environment‐friendly synthesis and decomposition. (For example, facile removal of lithium from organic redox molecule has been demonstrated.^[30]^) Furthermore, an appropriate molecular design should enable the fixation of organic redox molecules in the fabricated electrode.

**Figure 1:**
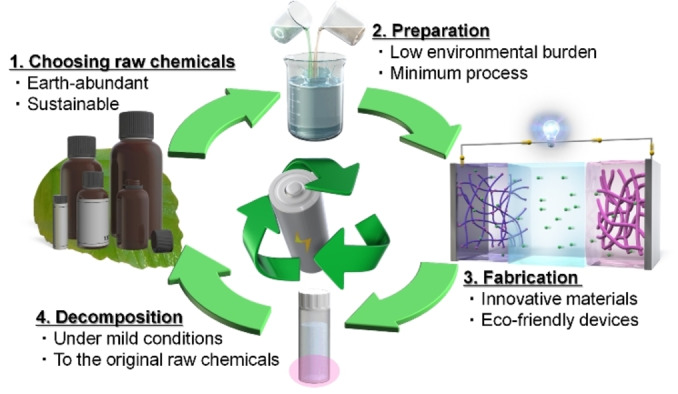
Sustainable and environment‐friendly recycling method for functional organic materials.

The present study aimed to demonstrate a sustainable and environment‐friendly recycling method for functional organic materials, as shown in Figure 1. We report that organic redox molecules could be prepared from earth‐abundant and sustainable raw materials (Figure 1, Step 1, the raw chemical requirements) via synthetic routes with low environmental burden (Figure 1, Step 2, the preparation requirements). An eco‐friendly and all‐organic battery was fabricated using organic redox molecules as both anode‐ and cathode‐active materials and using neutral aqueous solution as an electrolyte (Figure 1, Step 3, the fabrication requirements). After use, these electrode‐active materials werec separated and decomposed to their original raw chemicals under mild conditions (Figure 1, Step 4, the decomposition requirements).

## Results and Discussion

### Exploring and Designing Electrode‐Active Organic Molecules

To develop a sustainable and environment‐friendly recycling method for functional organic materials, we designed appropriate organic redox molecules using earth‐abundant and/or sustainable raw chemicals (Figure 1, Step 1) via a synthetic process with low environmental burden (Figure 1, Step 2). In addition, materials that could be decomposed into their original raw chemicals via a mild and process were selected (Figure 1, Step 4).

**Figure 2:**
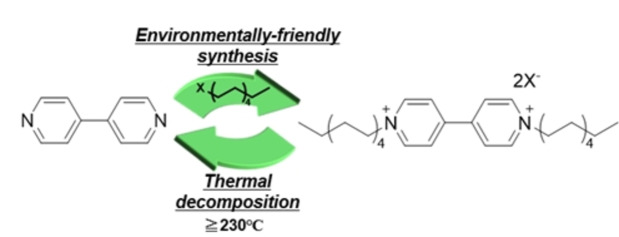
Synthesis and thermal decomposition of viologen derivatives. Viologen derivative was 1,1′‐didecyl‐4,4′‐bipyridinium dibromide (X: Br), which had a short alkyl chain to give appropriate water insolubility for viologen derivatives (other viologen derivatives are compared in Table S2). After its utilisation in aqueous solution, including much chloride ion, the counter‐ion of viologen derivative (X^−^) should become chloride ion.

After screening several organic redox molecules[^9^, ^31^] (major organic redox molecules are summarised in Figure S1), we focused on nitrogen heterocyclic compounds, such as viologen[^32^, ^33^] derivatives, which exhibit reversible redox capability in aqueous electrolytes (Figure 1, Step 3). These compounds could be prepared from biomolecules used as raw chemicals thereby meeting the raw chemical requirement mentioned in Figure 1, Step 1. (Please note that in cases where raw materials have already been reported to be earth‐abundant and sustainable, the raw materials may have been purchased from reagent companies in the current work.) Their physical characteristics (e.g., water solubility) were adjusted by changing the alkyl group connected with nitrogen atoms, via a facile reaction with bromo compounds (nucleophilic substitution reaction), thereby meeting the preparation requirements (Figure 1, Step 2). After functionalisation, they were decomposed via a mild and process (e.g., heating at >200 °C), thus, meeting the decomposition requirements mentioned in Figure 1, Step 4 (also see Figure 2). Although methyl viologen (1,1’‐dimethyl‐4,4’‐bipyridinium dichloride) are well‐known cytotoxic,[^34^, ^35^] these derivatives with functionalized and/or longer alkyl chains have possibly decreased their toxic[^34^, ^35^] and have been aggressively researched to be used in redox flow batteries.[^32^, ^36^, ^37^, ^38^] Therefore, the derivatives have the potential to meet the fabrication requirements mentioned in Figure 1, Step 3. We functionalised 4,4’‐bipyridine (raw chemical) with common alkylamines (heptane, decane, and dodecane) to prepare viologen derivatives and to prevent their dissolution in aqueous electrolytes (the synthetic routes for viologen derivatives are described in the Supporting Information Sections 1.3–1.5 and Table S2. These synthesis routes were based on previous papers[^32^, ^36^, ^37^]). We found that 1,1′‐didecyl‐4,4′‐bipyridinium dibromide had a short alkyl chain that resulted in appropriate water insolubility for viologen derivatives. Furthermore, a high yield of 1,1′‐didecyl‐4,4′‐bipyridinium dibromide (88 %) was prepared from 4,4’‐bipyridine and 1‐bromodecane in γ‐valerolactone by heating at 120 °C (Figure 2, Experimental details are provided in Supporting Information Section 1.6). 4,4’‐Bipyridine could be prepared from pyridine, which is present in alkaloid natural products and vitamins, and therefore can be sustainably synthesised via the biosynthetic route.^[39]^ Therefore, the raw chemical requirements, mentioned in Figure 1, Step1, were met. 1‐Bromodecane was synthesised from an alkane with multiple carbon atoms and a high boiling point and is usually categorised as an environment‐friendly organic solvent from the perspective of its life‐cycle assessment.[^40^, ^41^] γ‐Valerolactone can be easily derived from cellulose or hemicellulose and therefore from non‐food biomass, and is categorised as a promising green solvent.^[42]^ In addition to the facile purification process of 1,1′‐didecyl‐4,4′‐bipyridinium dibromide that only involves washing with water and vacuum drying, the synthetic route (Figure 2) has a low environmental burden, thus, meeting the preparation requirements mentioned in Figure 1, Step 2 (Experimental details are provided in Supporting Information Section 1.6).

The thermal analysis of 1,1′‐didecyl‐4,4′‐bipyridinium dibromide indicated a decomposition temperature of ≥230 °C (Figure S2a). ^1^H nuclear magnetic resonance (NMR) spectroscopy of 1,1′‐didecyl‐4,4′‐bipyridinium dibromide revealed that after heating at 230 °C for 24 h, it had completely decomposed to 4,4’‐bipyridine (Figure S3). In addition, 1,1′‐didecyl‐4,4′‐bipyridinium dichloride showed the same behaviour (Figure S2b and S4). These results indicated that the synthesised 1,1′‐didecyl‐4,4′‐bipyridinium dibromide and 1,1′‐didecyl‐4,4′‐bipyridinium dichloride were decomposed at 230 °C to the original raw chemical, 4,4’‐bipyridine, thus, meeting the decomposition requirements mentioned in Figure 1, Step 4 (experimental and purification details are presented in the Supporting Information Sections 1.12–1.13).

Subsequently, the suitability of the viologen derivative as an electrode‐active material in neutral aqueous solution (e.g., 3 M KCl aqueous electrolyte) was investigated to determine whether they could meet the fabrication requirements mentioned in Figure 1, Step 3. To study its electrochemical properties, we prepared a slurry of the viologen derivative mixed with carbon nanotubes (30 wt% of total weight) as the binder‐free conductive additive in dihydrolevoglucosenone (cyrene^TM^), and coated the slurry on a glassy carbon plate or a graphite foil to fabricate the electrode (further details provided in Supporting Information Section 1.8). Carbon nanotubes are one of the promising binder‐free conductive additives,^[43]^ which facilitate separation of organic redox molecules from the fabricated electrode, and a reduction of the decomposition process post‐utilisation, thereby meeting the decomposition requirements mentioned in Figure 1, Step 4. Furthermore, a method to manufacture carbon nanotubes from waste plastic has been developed,^[44]^ and therefore carbon nanotubes can help to overcome the challenge of plastic pollution. Cyrene^TM^ is a biological, bio‐renewable, and green solvent, and is an environment‐friendly alternative to harmful organic solvents, such as *N*,*N*‐dimethylformamide and *N*‐methylpyrrolidone.[^45^, ^46^, ^47^] Collectively, these observations indicated that the developed electrode‐fabrication process was eco‐friendly, thus, meeting the fabrication requirements in Figure 1, Step 2.

**Figure 3:**
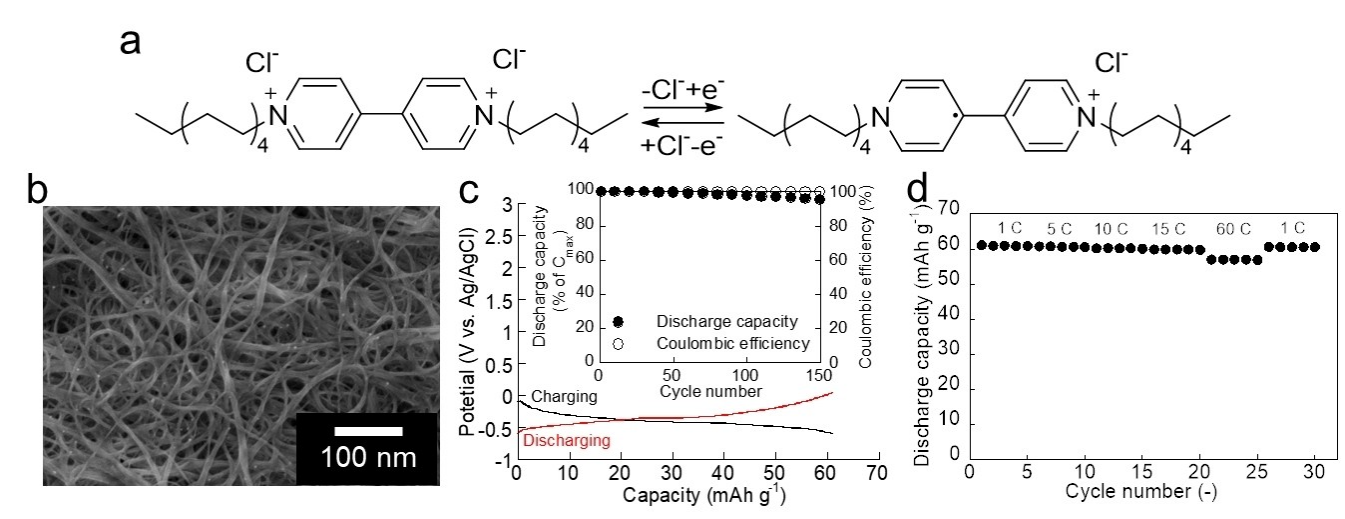
Electrochemical properties of the organic redox molecules. a) Redox scheme of the viologen/carbon nanotube composite electrode (viologen: 1,1′‐didecyl‐4,4′‐bipyridinium dibromide or 1,1′‐didecyl‐4,4′‐bipyridinium dichloride). b) Scanning electron microscope (SEM) image of the viologen electrode. c) Charging (black)/discharging (red) curves of the viologen electrode (15 C) in 3 M KCl aqueous electrolyte. Inset: Cycle test of the electrode (15 C). d) Rate capability of the viologen electrode (1, 5, 10, 15, and 60 C).

Scanning electron microscope analysis of the fabricated electrodes demonstrated that 1,1′‐didecyl‐4,4′‐bipyridinium dibromide (Figure 3b) was dispersed on the carbon nanotube. The electrode charging‐discharging curves of 1,1′‐didecyl‐4,4′‐bipyridinium dibromide (Figure 3c) exhibited plateau voltages, like redox polymers,^[48]^ at −0.3 V vs. Ag/AgCl, and its c(discharging capacity/charging capacity) were approximately 99 %. Based on the findings of previous studies of viologen derivatives,[^32^, ^33^, ^49^, ^50^, ^51^] the redox reaction was attributed to the one‐electron reduction of 1,1′‐didecyl‐4,4′‐bipyridinium dibromide (Figure 3a). Furthermore, after electrochemical tests in 3 M KCl aqueous electrolyte, the counteranion of 1,1′‐didecyl‐4,4′‐bipyridinium was found to be a chloride ion. Its capacity was 60.1 mAh/g_molecule_ for 1,1′‐didecyl‐4,4′‐bipyridinium dibromide (98 % of the theoretical capacity of 61.1 mAh/g_molecule_), suggesting that almost all organic redox molecules contributed to the charge storage (It might be noted that previous reports have shown that the carbon nanotubes contribute little to capacity[^11^, ^52^, ^53^]). Its cycle test (Figure 3c Inset) revealed that 96 % of the initial capacity for 1,1′‐didecyl‐4,4′‐bipyridinium dibromide was maintained after 100 cycles, thus, demonstrating the robustness of the fabricated electrode of the organic redox molecule similar to that of redox polymers.[^9^, ^54^] The rate performances of the discharging process are shown in Figure 3d. The electrode maintained almost full discharging capacities even at rapid discharging (15 C), which corresponded to full discharging within 240 s. These results clearly demonstrated that the organic redox molecule was suitable as an electrode‐active material in the neutral aqueous electrolyte, thereby meeting the fabrication requirements mentioned in Figure 1, Step 2.

### Development of an Eco‐Friendly & All‐Organic Battery and its Electrode‐Active Material′s Decomposition After Use

**Figure 4:**
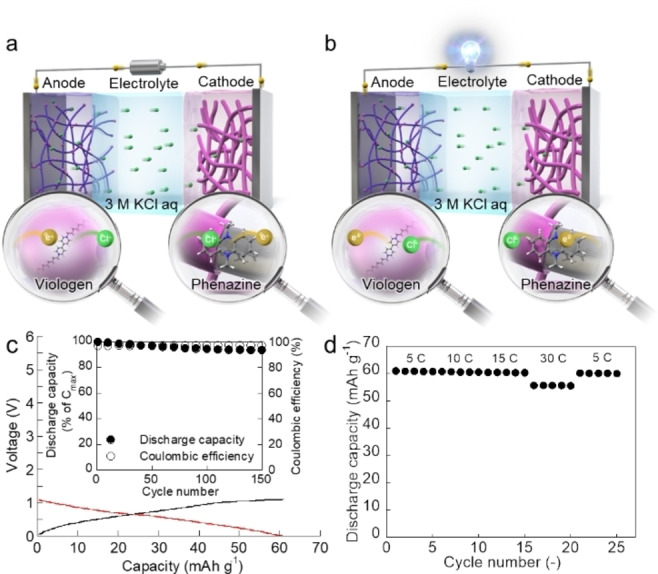
An eco‐friendly and all‐organic battery developed using organic redox molecules and neutral aqueous electrolyte. Schematic showing the all‐organic battery. a) Charging. b) Discharging. c) Charging (black)/discharging (red) of the battery (15 C). Inset: Cycle test of the battery (15 C). d) Rate capability of the battery (5, 10, 15, and 30 C).

The eco‐friendly and all‐organic battery was fabricated using the 1,1′‐didecyl‐4,4′‐bipyridinium dibromide/carbon composite electrode as an anode, 5,10‐dihydro‐5,10‐dimethylphenazine/carbon composite electrode as a cathode, and 3 M KCl aqueous solution as an electrolyte (Figures 4a and 4b). 5,10‐Dihydro‐5,10‐dimethylphenazine is the representative phenazine derivative, and phenazines are components of biomolecules involved in metabolism, and therefore they are possibly and sustainably prepared via the biosynthetic route[^55^, ^56^] similar to the raw chemicals of viologen derivatives. Furthermore, 5,10‐dihydro‐5,10‐dimethylphenazine exhibits low water solubility and has redox capability at a moderate positive potential at 3.25 V vs. Li/Li^+[49–51,57]^ (electrochemical properties’ details are presented in the Supporting Information Section 1.11 and Figure S1), and does not require further functionalisation and decomposition, simultaneously meeting the requirements for raw chemical, preparation, and decomposition, mentioned in Figure 1. Thus, we chose 5,10‐dihydro‐5,10‐dimethylphenazine as a cathode‐active material.

Only the chloride ion (Cl^−^) in the electrolyte is transported between the cathode and anode during charging/discharging to compensate for the charge neutrality of these electrodes.^[58]^ Therefore, the developed organic battery is a rocking‐chair battery and exhibited a voltage of 0.5 V (Figure 4c) and high coulombic efficiency (≧97 %). (Figure 4c Inset), even in open‐air conditions, thereby demonstrating reversible charge storage capability. The anode capacity used in the battery was 60.3 mAh/g_molecule_, suggesting that 1,1′‐didecyl‐4,4′‐bipyridinium dibromide almost completely stored charges. The rate performance of the discharging process (Figure 4d) showed that the battery maintained almost full discharging capacity even at rapid discharging of 15 C. Furthermore, the battery exhibited high cyclability (>95 %) after 100 cycles, which was similar to or higher than those of state‐of‐the‐art all‐organic batteries previously developed using neutral aqueous electrolytes and organic redox polymers (see Table S1).[^14^, ^15^, ^16^, ^17^, ^18^, ^59^] Considering this slight degradation, since there was no dissolution of electrode‐active materials during the cycles, the electrode‐active material (especially the cathode‐active electrode) may become electrochemically inactive for some reason. Since NMR measurements of the electrodes after electrochemical measurements showed no structural changes in the electrode‐active materials, the cause is presumably that the electrode‐active material lost contact with the conductive additive or electrolyte during the cycles. In our continuous work, we will improve the cyclability by optimizing the electrode structure. Although the developed organic battery requires further improvements (especially voltage and capacity towards practical use), these results conceptually demonstrated that these organic redox molecules met the fabrication requirements mentioned in Figure 1.

Due to the simple configuration of the battery whereby electrodes are simply immersed in the electrolyte, the battery can be easily decomposed into anode, cathode, and electrolyte. The organic redox molecule/carbon composite electrode can also be easily scraped off the current collector. When immersed in ethanol and shaken for a few minutes, the composite electrode consisting of 1,1′‐didecyl‐4,4′‐bipyridinium dichloride was completely separated from the carbon nanotubes at a high yield (>97 %) (Figure S11, further details provided in Supporting Information Section 1.14, it might be noted that 5,10‐dihydro‐5,10‐dimethylphenazine was also separated in a high yield of >90 %; the separated material could be reused as is). 1,1′‐Didecyl‐4,4′‐bipyridinium dichloride (or 1,1′‐didecyl‐4,4′‐bipyridinium dibromide) was decomposed by heating at 230 °C for 24 h without the production of some sort of polymeric or polycondensate structure. After a purification process (using a silica gel column with methyl tetrahydrofuran, which is a green solvent), the original raw chemical of 4,4′‐bipyridine was obtained at a high yield (approximately 80 %, see Supporting Information Section 1.15). These results demonstrated that these organic redox molecules met the decomposition requirements mentioned in Figure 1, Step 4. Furthermore, we confirmed that 1,1’‐didecyl‐4,4’‐bipyridinium dibromide could be regenerated from the decomposition product 4,4’‐bipyridine, and it reproduced the electrochemical properties (as shown in Figure 3b). Thus, a sustainable and environment‐friendly recycling method for functional organic materials was developed as shown in Figure 1.

## Conclusions

This study demonstrated a sustainable and environment‐friendly recycling method example for functional organic materials in Figure 1. We showed that organic redox molecules as a solid‐state electrode‐active material in aqueous electrolyte are a potential new clean and sustainable option to conventional organic redox materials. We also demonstrated eco‐friendly battery, which uses organic redox molecules as anode‐ and cathode‐active materials and neutral aqueous solution as an electrolyte, using a simple beaker cell as the most basic battery configuration. Although the developed organic battery requires further improvements, this study presents a first step for developing advanced functional organic redox materials that are both sustainable and environment‐friendly.

## Conflict of Interests

The authors declare no conflict of interest.

1

## Supporting information

As a service to our authors and readers, this journal provides supporting information supplied by the authors. Such materials are peer reviewed and may be re‐organized for online delivery, but are not copy‐edited or typeset. Technical support issues arising from supporting information (other than missing files) should be addressed to the authors.

Supporting Information

## Data Availability

The data that support the findings of this study are available from the corresponding author upon reasonable request.
