# Supplementary material for: Recyclable Organic Redox Molecules for Sustainable Batteries
Source: ChemSusChem. 2025 Mar 31;18(11):e202402707. doi: 10.1002/cssc.202402707 (PMC12131672; doi:10.1002/cssc.202402707)
Supplement: Supplementary file 1 — Supporting Information [file CSSC-18-e202402707-s001.pdf]

ChemSusChem

Supporting Information

Recyclable Organic Redox Molecules for Sustainable Batteries

Kouki Oka* and Hitoshi Kasai

Recyclable Organic Redox Molecules for Sustainable Batteries

Kouki Oka[a,b,c]* and Hitoshi Kasai[a]

[a] Prof. Dr. K. Oka and Prof. Dr. H. Kasai
Institute of Multidisciplinary Research for Advanced Materials
Tohoku University
2-1-1 Katahira, Aoba-ku, Sendai, Miyagi 980-8577, Japan
E-mail: oka@tohoku.ac.jp (Kouki Oka)

[b] Prof. Dr. K. Oka
Carbon Recycling Energy Research Center
Ibaraki University
4-12-1 Nakanarusawa, Hitachi, Ibaraki 316-8511, Japan
E-mail: oka@tohoku.ac.jp (Kouki Oka)

[c] Prof. Dr. K. Oka
Deuterium Science Research Unit, Center for the Promotion of Interdisciplinary Education and Research
Kyoto University
Yoshida, Sakyo-ku, Kyoto 606-8501, Japan
E-mail: oka@tohoku.ac.jp (Kouki Oka)

1. Supporting methods

1.1. Materials.

4,4'-Bipyridyl, 1-bromoheptane, 1-bromodecane, 1-bromododecane, and tetrabutylammonium chloride were purchased from Tokyo Chemical Industry Co. Ammonium hexafluorophosphate was purchased from FUJIFILM Wako Pure Chemical Co. Acetonitrile and other chemicals were obtained from Kanto Chemical Co. Cyrene^{TM}, 2-methyltetrahydrofuran (inhibitor-free) and γ-valerolactone were purchased from Sigma-Aldrich Co. LCC.

1.2. Measurements.

^{1}H NMR spectra were recorded on a JEOL ECZ400YH spectrometer with chemical shifts downfield from tetramethylsilane as the internal standard. Thermal analyses were performed using a Rigaku ThermoPlus EVO2 TG8121 at a heating rate of 5 °C min^{-1} under nitrogen.

1.3. Synthesis of 1,1′-diheptyl-4,4′-bipyridinium dibromide.

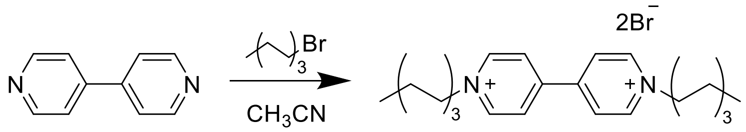

Scheme S1. Synthesis of 1,1′-diheptyl-4,4′-bipyridinium dibromide.

1,1′-Diheptyl-4,4′-bipyridinium dibromide was synthesized following the previous report[1]. 4,4'-Bipyridyl (0.156 g, 1.0 mmol) and 1-bromoheptane (0.63 mL, 4.0 mmol) were refluxed in acetonitrile (5 mL) for 24 hours. After reaction, resulting yellow mixture was filtered, was washed by acetone, and was dried to yield as a yellow solid (0.252 g, 0.49 mmol, 49 %). 1,1′-Diheptyl-4,4′-bipyridinium dibromide was soluble in cyrene™, dimethyl sulfoxide (DMSO), methanol, γ-valerolactone, and water. ^{1}H NMR (400 MHz, D_2O, δ) in Figure S5: 8.99 (d, J = 7.33 Hz, 4H), 8.42 (d, J = 6.41 Hz, 4H), 4.61 (t, J_1 = 6.87, J_2 = 7.79 Hz, 4H), 1.97 (m, 4H), 1.26 (m, 8H), 1.12 (m, 8H), 0.74 (t, J_1 = 6.87, J_2 = 7.73 Hz, 6H).

1.4. Synthesis of 1,1′-didecyl-4,4′-bipyridinium dibromide.

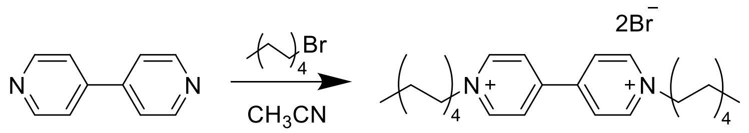

Scheme S2. Synthesis of 1,1′-didecyl-4,4′-bipyridinium dibromide.

1,1′-Didecyl-4,4′-bipyridinium dibromide was synthesized following the previous report[2]. 4,4'-Bipyridyl (0.156 g, 1.0 mmol) and 1-bromodecane (0.82 mL, 4.0 mmol) were refluxed in acetonitrile (5 mL) for 24 hours. After reaction, resulting yellow mixture was filtered, was washed by acetonitrile and diethyl ether, and was dried to yield 1,1′-didecyl-4,4′-bipyridinium dibromide as a yellow solid (0.320 g, 0.53 mmol, 53 %). 1,1′-Didecyl-4,4′-bipyridinium dibromide was soluble in cyrene™, dimethyl sulfoxide (DMSO), methanol, and γ-valerolactone. ^{1}H NMR (400 MHz, DMSO-d_6, δ) in Figure S6: 9.35 (d, J = 6.87 Hz, 4H), 8.75 (d, J = 6.41 Hz, 4H), 4.64 (t, J_1 = 7.33 Hz, J_2 = 7.33 Hz, 4H), 1.94 (m, 4H), 1.24 (m, 28H), 0.81 (t, J_1 = 6.87 Hz, J_2 = 6.87 Hz, 6H).

1.5. Synthesis of 1,1′-didodecyl-4,4′-bipyridinium dibromide.

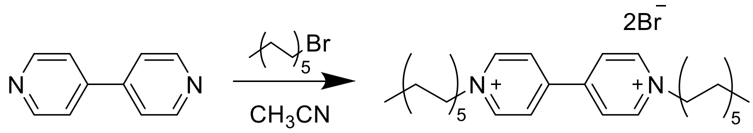

Scheme S3. Synthesis of 4,4'-bipyridinium-1,1'-didodecyl-bromide.

4,4'-Bipyridinium-1,1'-didodecyl-bromide was synthesized following the previous report[2]. 4,4'-Bipyridyl (0.155 g, 1.0 mmol) and 1-bromododecane (0.95 mL, 4.0 mmol) were refluxed in acetonitrile (5 mL) for 24 hours. After reaction, resulting yellow mixture was filtered, was washed by acetonitrile and diethyl ether, and was dried to yield 1,1′-didodecyl-4,4′-bipyridinium dibromide as a yellow solid (0.554 g, 0.85 mmol, 85 %). 1,1′-Didodecyl-4,4′-bipyridinium dibromide was soluble in cyrene™, dimethyl sulfoxide (DMSO), methanol, and *γ*-valerolactone. ^{1}H NMR (400 MHz, DMSO-d_6, δ) in Figure S7: 9.36 (d, J = 6.87 Hz, 4H), 8.76 (d, J = 6.87 Hz, 4H), 4.65(t, J_1 = 7.33 Hz, J_2 = 7.33 Hz, 4H), 1.93 (m, 4H), 1.23 (m, 36H), 0.81 (t, J_1 = 6.41 Hz, J_2 = 6.87 Hz, 6H).

1.6. Synthesis of 1,1′-didecyl-4,4′-bipyridinium dibromide in *γ*-valerolactone.

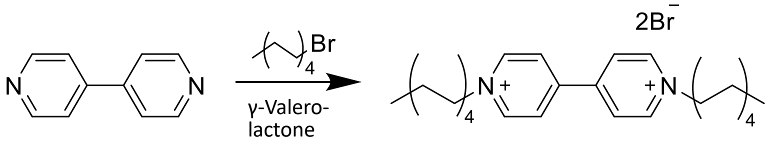

Scheme S4. Synthesis of 1,1′-didecyl-4,4′-bipyridinium dibromide in *γ*-valerolactone.

4,4'-Bipyridyl (0.156 g, 1.0 mmol) and 1-bromodecane (0.83 mL, 4.0 mmol) were heated to 120℃ in *γ*-valerolactone (5 mL) for 24 hours. After the reaction, resulting yellow mixture was filtered, and was washed with water. After vacuum drying, 1,1′-didecyl-4,4′-bipyridinium dibromide was yielded as a yellow solid (0.528 g, 0.88 mmol, 88 %). 1,1′-Didecyl-4,4′-bipyridinium dibromide was soluble in cyrene™, dimethyl sulfoxide (DMSO), methanol, and *γ*-valerolactone. ^{1}H NMR (400 MHz, DMSO-d_6, δ) in Figure S8: 9.36 (d, J = 5.95 Hz, 4H), 8.76 (d, J = 6.41 Hz, 4H), 4.65 (t, J_1 = 7.33, J_2 = 7.73 Hz , 4H), 1.94 (m, 4H), 1.24 (m, 28H), 0.82 (t, J_1 = 5.50, J_2 = 6.87 Hz , 4H).

1.7. Preparation of 1,1′-didecyl-4,4′-bipyridinium dichloride.

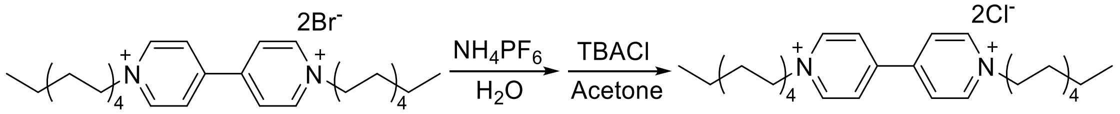

Scheme S5. Synthesis of 1,1′-didecyl-4,4′-bipyridinium dichloride.

1,1′-Didecyl-4,4′-bipyridinium dibromide was dissolved in DMSO (20 mL). Then the solution was added to aqueous ammonium hexafluorophosphate solution (0.61 M, 10 mL), and the mixed solution was stirred for 5 min. The resulting mixture was filtered, was washed with water, and was dried to yield a white powder. Then, the white powder was dissolved in acetone (10 mL). The solution was added to acetone solution including tetrabutylammonium chloride (0.60 M, 10 mL), and was stirred for 5 min. The resulting mixture was filtered, was washed with acetone, and then was dried to yield a white powder, which was characterized as 1,1′-didecyl-4,4′-bipyridinium dichloride (0.184 g, 0.36 mmol, 68 %), as below. ^{1}H NMR (400 MHz, DMSO-d_6, δ) in Figure S9: 9.35 (d, J = 6.87 Hz, 4H), 8.75 (d, J = 6.41 Hz, 4H), 4.64 (t, J_1 = 7.33 Hz, J_2 = 7.33 Hz, 4H), 1.94 (m, 4H), 1.24 (m, 28H), 0.81 (t, J_1 = 6.87 Hz, J_2 = 6.87 Hz, 6H). Anal. calcd for $C_{30}H_{50}Cl_2N_2$: C 70.7, H, 9.9; found: C 70.5, H 10.0. 1,1′-Didecyl-4,4′-bipyridinium dichloride was soluble in cyrene™, dimethyl sulfoxide (DMSO), methanol, and γ-valerolactone.

1.8. Electrode preparation.

The organic redox molecule/carbon nanotube composite electrodes were prepared by coating the slurry of the organic redox molecule, single-walled carbon nanotube (SWNT) (7:3 in w/w), and cyrene^{TM} onto glassy carbon substrates. The polymer carbon composite was coated on a glassy carbon plate or a graphite foil. The mass loading of the organic redox molecule was adjusted to ca. 1.0 mg/cm^2. The composite electrode was heated on a hotplate at 120°C for 12 h to ambient atmosphere to remove cyrene^{TM}.

1.9. Electrochemical measurements.

The electrochemical measurements were performed with 3 M KCl aqueous solution as the electrolyte in air. A potentiostat system (HOKUTO DENKO CORPORATION, HZ-7000) was applied to all electrochemical measurements. A carbon disk and coiled platinum wire were used as the working and counter electrodes, respectively, and the working potential was measured vs. an Ag/AgCl reference electrode. The same electrochemical measurements were conducted at least five times.

1.10. Battery evaluation.

A tailor-made beaker cell (20 cm^3 electrolyte) as reported in our previous papers[3] was employed as the electrochemical cell. Separation of the compartments was achieved using a fine glass filter, allowing only the electrolyte to pass during the measurement. Both anode and cathode sections were filled with 3 M KCl aqueous solution left open to the air. The battery evaluation tests using similarly fabricated batteries were performed at least 5 times.

1.11. Characterization of the phenazine/carbon composite electrode.

Scanning electron microscope analysis of the fabricated electrode demonstrated that 5,10-dihydro-5,10-dimethylphenazine (Figure S10b) was covered on the carbon nanotube. The electrode charging-discharging curves of 5,10-dihydro-5,10-dimethylphenazine (Figure S10c) exhibited plateau voltage at +0.2 V vs. Ag/AgCl, and its coulombic efficiency (discharging capacity/charging capacity) was approximately 98%. Based on the findings of previous studies[4], the redox reaction was attributed to the one-electron oxidation of 5,10-dihydro-5,10-dimethylphenazine (Figure S10a). The capacity was 121 mAh/$g_{molecule}$ (95% of the theoretical capacity of 127 mAh/$g_{molecule}$), suggesting that almost all organic redox molecules contributed to the charge storage. Their cycle test (Figure S10c Inset) revealed that 94% of the initial capacity for 5,10-dihydro-5,10-dimethylphenazine was maintained after 100 cycles, thus, demonstrating the robustness of the fabricated electrode. The rate performances of the discharging process are shown in Figure S10d. The electrode maintained almost full discharging capacities even at rapid discharging (15 C), which corresponded to full discharging within 240 s. These results clearly demonstrated that 5,10-dihydro-5,10-dimethylphenazine was suitable as an electrode-active material in the neutral aqueous electrolyte.

1.12. Decomposition of 1,1′-didecyl-4,4′-bipyridinium dibromide.

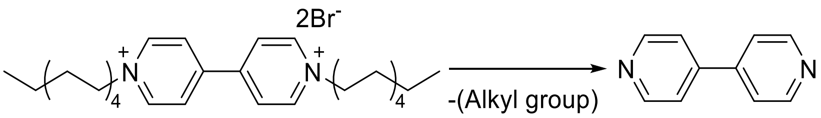

Scheme S6. Decomposition of 1,1′-didecyl-4,4′-bipyridinium dibromide.

1,1′-Didecyl-4,4′-bipyridinium dibromide (9.9 mg) was heated at 230 °C for 24 h (heating rate: 5 °C min^{-1}, gas (N_2) flow: 50 mL/min), was purified using column chromatography on silica gel with methyltetrahydrofuran as eluents, and then the solvent was evaporated. After sublimation purification to remove 1-bromododecane and a compound with only one alkyl chain remaining, 4,4'-bipyridine yielded a high yield of 78%. The ^{1}H-NMR spectra were almost the same as the case of 1,1′-didecyl-4,4′-bipyridinium dichloride, as summarized in Figure S3. Anal. calcd for $C_{10}H_8N_2$: C 76.9, H, 5.2; found: C 76.8, H 4.2.

1.13. Decomposition of 1,1′-didecyl-4,4′-bipyridinium dichloride.

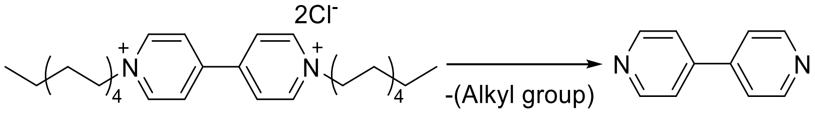

Scheme S7. Decomposition of 1,1′-didecyl-4,4′-bipyridinium dichloride.

1,1′-Didecyl-4,4′-bipyridinium dichloride (9.1 mg) was heated at 230 °C for 24 h (heating rate: 5 °C min^{-1}, gas (N_2) flow: 50 mL/min) , was purified using column chromatography on silica gel with methyltetrahydrofuran as eluents, and then the solvent was evaporated. After sublimation purification to remove 1-chlorododecane and a compound with only one alkyl chain remaining, 4,4'-bipyridine yielded a high yield of 81%. The ^{1}H-NMR spectra were summarized in Figure S4.

1.14. Separation of 1,1′-didecyl-4,4′-bipyridinium dichloride/carbon composite electrode to 1,1′-didecyl-4,4′-bipyridinium dichloride and carbon.

After electrochemical tests in 3 M KCl aqueous electrolyte, the counteranion of 1,1′-didecyl-4,4′-bipyridinium should be chloride ion. The fabricated electrode consisting of 1,1′-didecyl-4,4′-bipyridinium dichloride (81.6 mg) and carbon nanotube (30 wt% of total weight) was immersed in ethanol (20 mL) in a vessel and was shaken for 5 min. The solution was filtered and vacuum dried to yield 1,1′-didecyl-4,4′-bipyridinium dichloride in a high yield of 97 %. The composite electrode was simply composed of 1,1′-didecyl-4,4′-bipyridinium dichloride and carbon nanotube, and therefore the separated 1,1′-didecyl-4,4′-bipyridinium dichloride was high purity.

1.15. Decomposition of the used electrode composed of 1,1′-didecyl-4,4′-bipyridinium dichloride and carbon to the original raw mataeiral.

A used electrode consisting of 1,1′-didecyl-4,4′-bipyridinium dichloride (30.1 mg, calculated from the 1,1'-didecyl-4,4'-bipyridinium dibromide used for the fabrication of the composite electrode) and carbon nanotube was put in 20 mL of ethanol in a vessel and was shaken for 5 min. The solution was filtered and vacuum dried to give 1,1′-didecyl-4,4′-bipyridinium dichloride. Then, 1,1′-didecyl-4,4′-bipyridinium dichloride was heated at 230 °C for 24 h, and was purified using column chromatography on silica gel with methyltetrahydrofuran as eluents, to give the original raw chemical, 4,4'-bipyridine, in a high yield of 79%. When further purification was required, sublimation purification was performed.

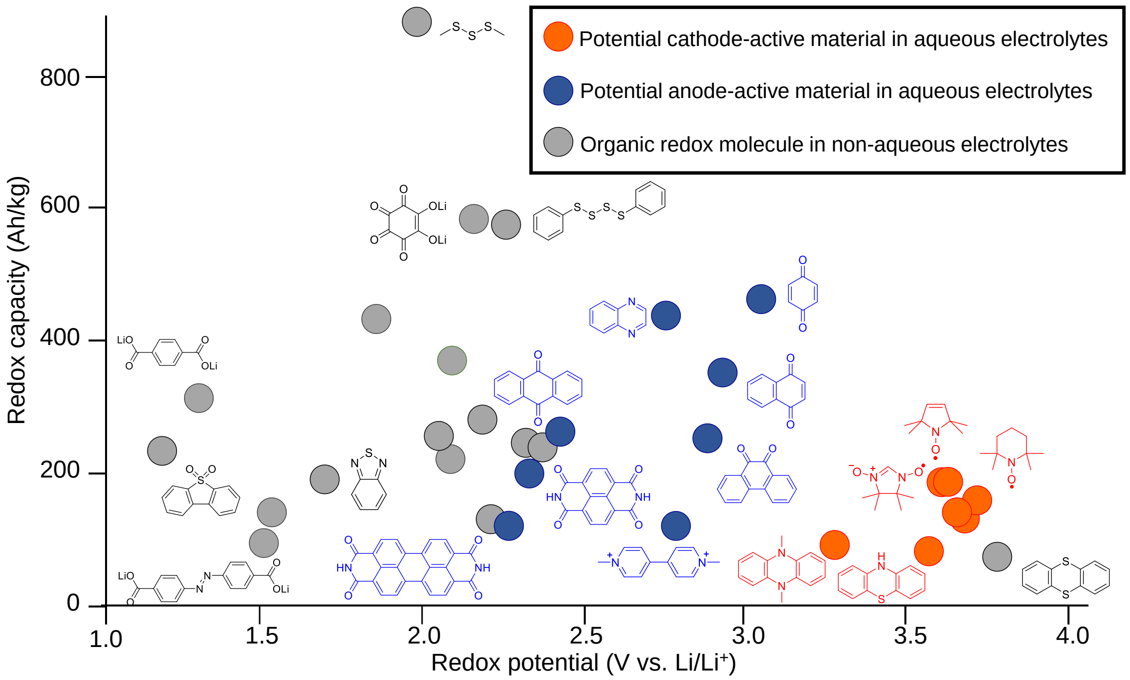

Figure S1. Summary of the major organic redox molecules[5].

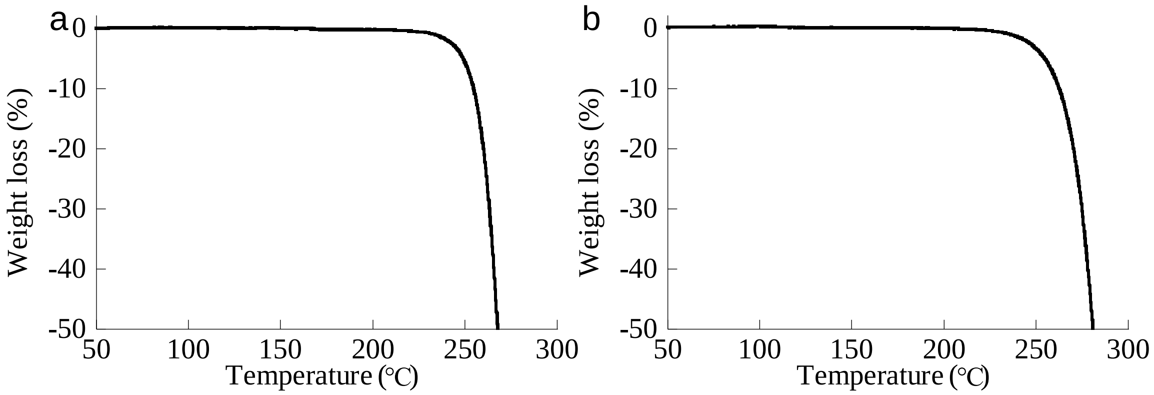

Figure S2. Thermalgravimetric analysis of 1,1′-didecyl-4,4′-bipyridinium dibromide and 1,1′-didecyl-4,4′-bipyridinium dichloride.

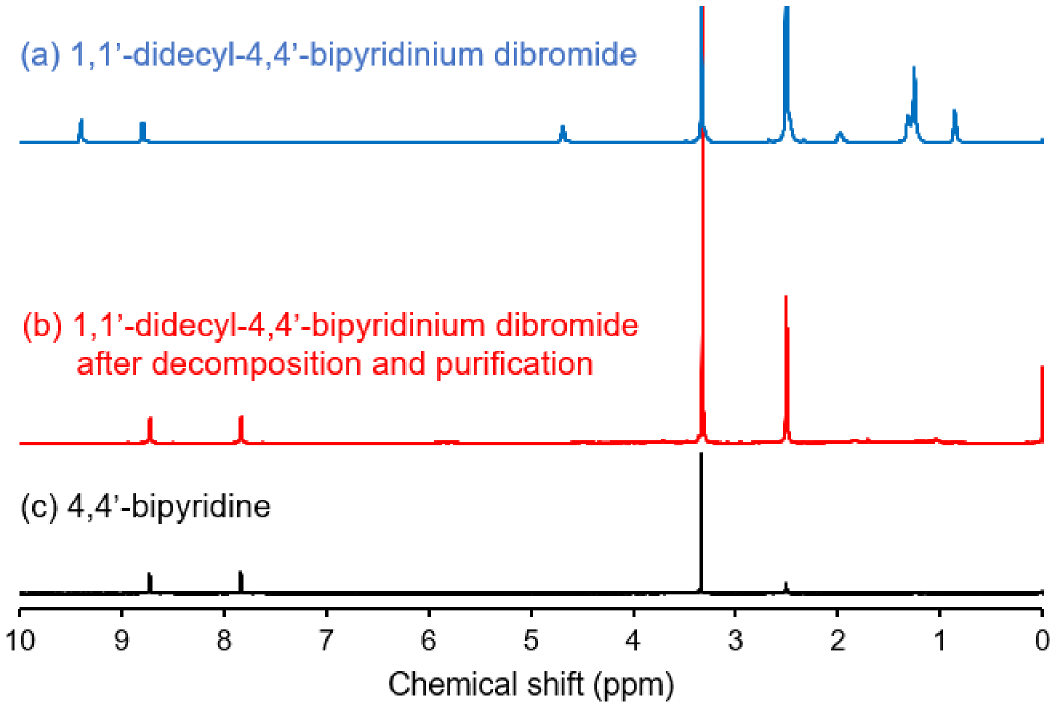

Figure S3. ^{1}H NMR spectra (dimethyl sulfoxide-d_6) of (a) 1,1′-didecyl-4,4′-bipyridinium dibromide, (b) 1,1′-didecyl-4,4′-bipyridinium dichloride after decomposition for 24 h and purification, and (c) 4,4′-bipyridine.

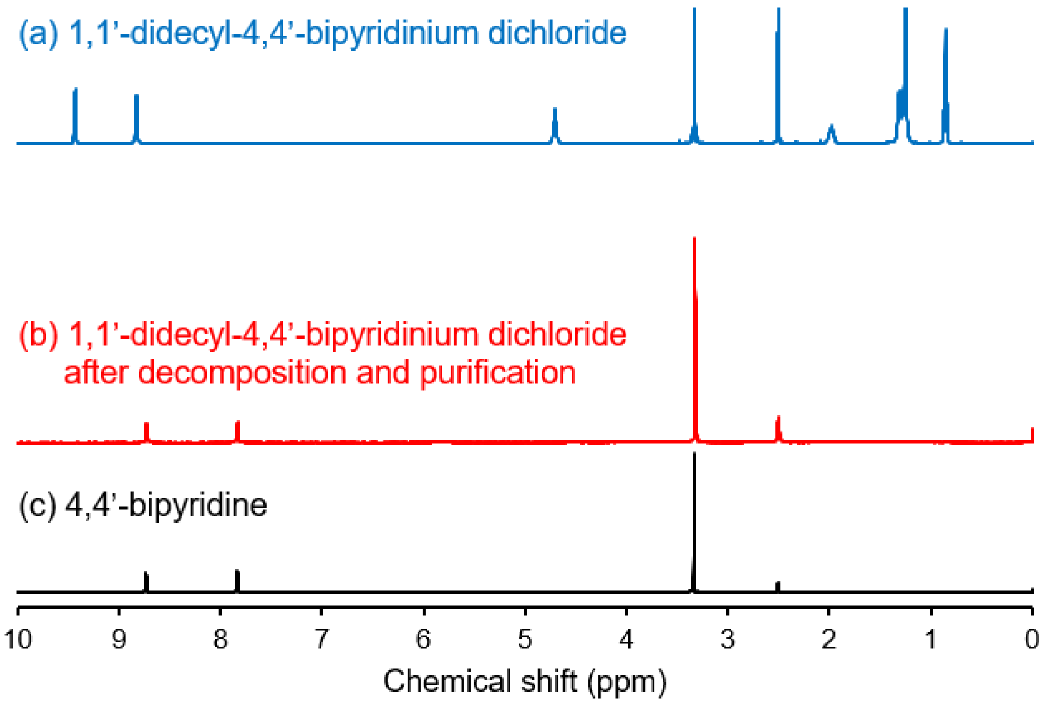

Figure S4. ^{1}H NMR spectra (dimethyl sulfoxide-d_6) of (a) 1,1′-didecyl-4,4′-bipyridinium dichloride, (b) 1,1′-didecyl-4,4′-bipyridinium dichloride after decomposition for 24 h and purification, and (c) 4,4′-bipyridine.

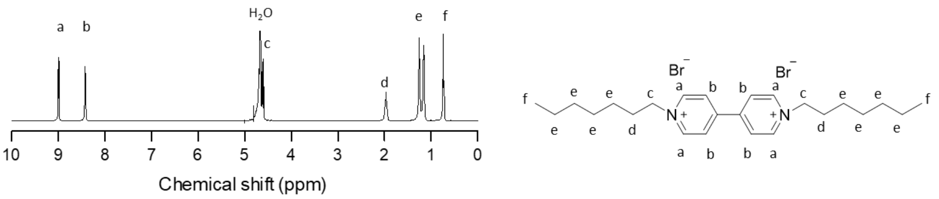

Figure S5. 400 MHz ^{1}H NMR spectrum (D_2O) of 1,1′-diheptyl-4,4′-bipyridinium dibromide.

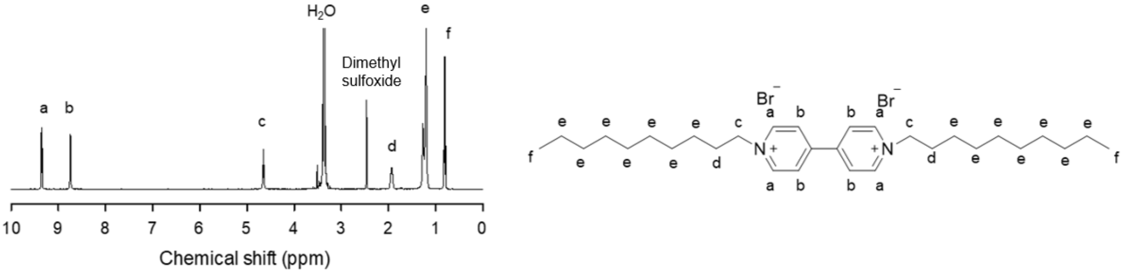

Figure S6. 400 MHz ^{1}H NMR spectrum (dimethyl sulfoxide-d_6) of 1,1′-didecyl-4,4′-bipyridinium dibromide.

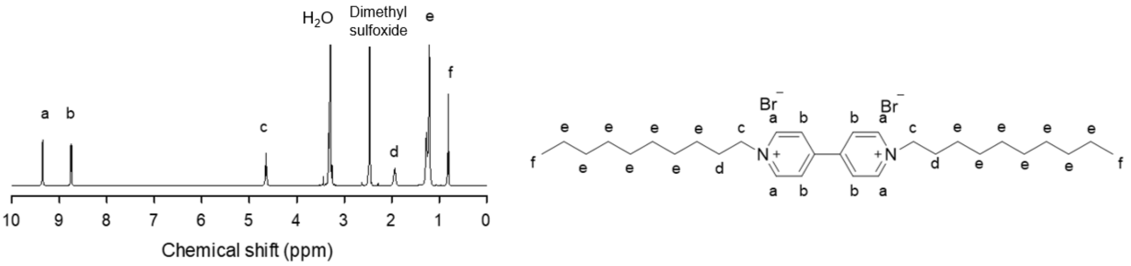

Figure S7. 400 MHz ^{1}H NMR spectrum (dimethyl sulfoxide-d_6) of 1,1′-didodecyl-4,4′-bipyridinium dibromide.

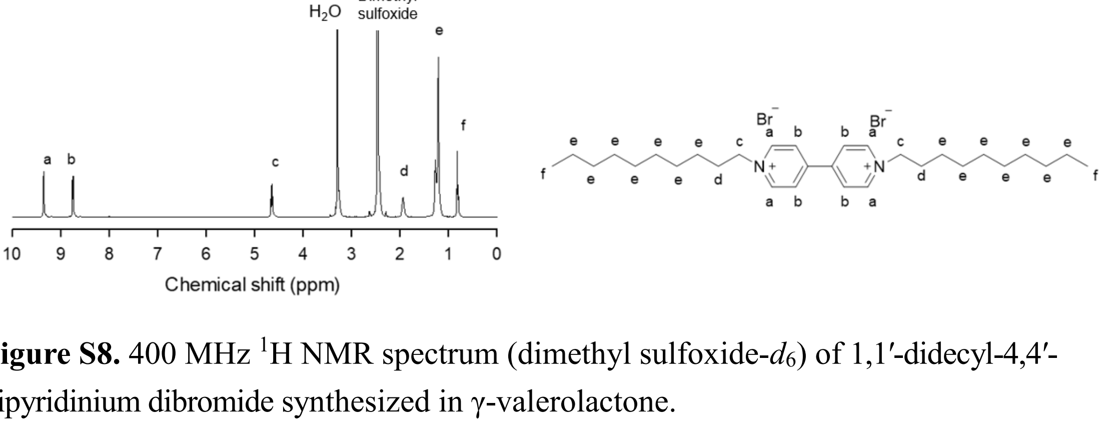

Figure S8. 400 MHz ^{1}H NMR spectrum (dimethyl sulfoxide-d_6) of 1,1′-didecyl-4,4′-bipyridinium dibromide synthesized in γ-valerolactone.

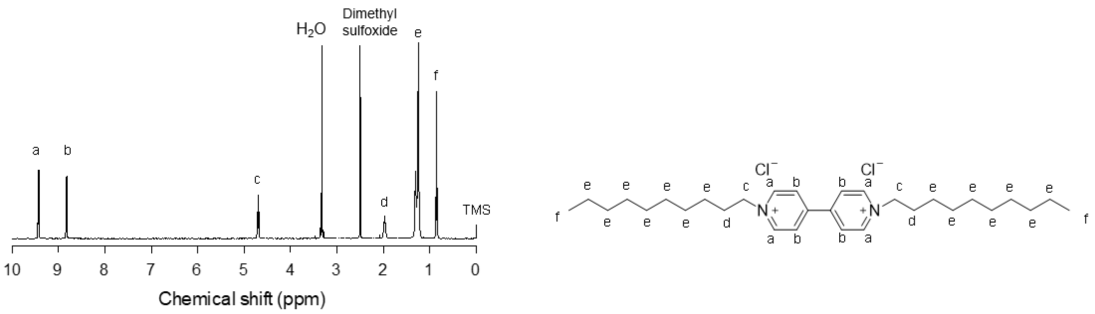

Figure S9. 400 MHz ^{1}H NMR spectrum (dimethyl sulfoxide-d_6) of 1,1′-didecyl-4,4′-bipyridinium dichloride.

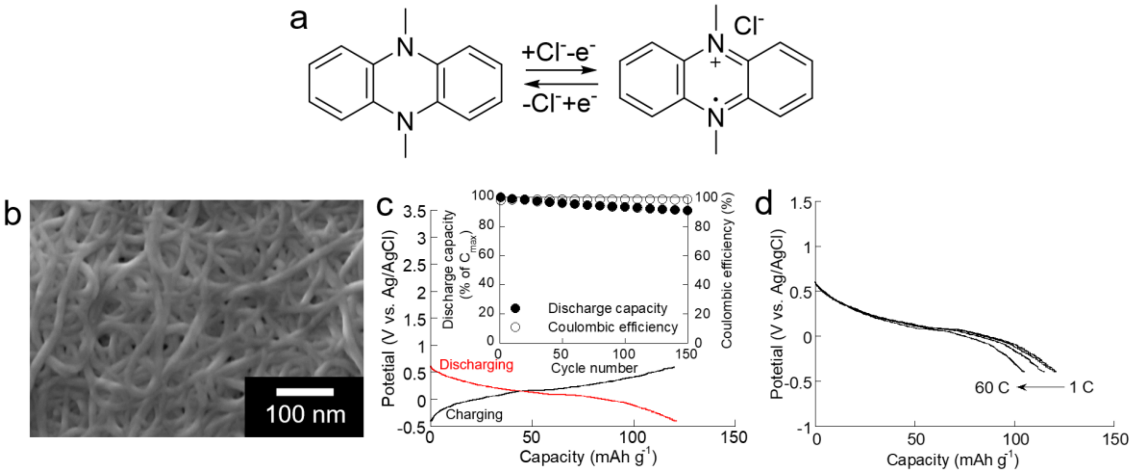

Figure S10. Electrochemical properties of the organic redox molecules. a) Redox scheme of the phenazine/carbon nanotube composite electrode (phenazine: 5,10-dihydro-5,10-dimethylphenazine). b) SEM image of the phenazine electrode. c) Charging (black)/discharging (red) curves of the phenazine electrode (15 C) in 3 M KCl aqueous electrolyte. Inset: Cycle test of the electrode (15 C). d) Rate capability of the phenazine electrode (1, 5, 10, 15, 30, and 60 C).

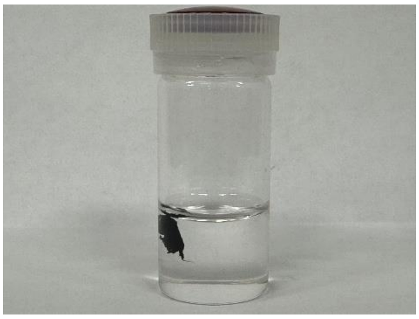

Figure S11. Photograph of the separation process of 1,1′-didecyl-4,4′-bipyridinium dichloride from composite electrode before shaking. When immersed in ethanol (1 mL) and shaken for a few minutes, the composite electrode (3.2 mg) consisting of 1,1′-didecyl-4,4′-bipyridinium dichloride was completely separated from the carbon nanotubes at a high yield (>97%).

Table S1. List of all-organic batteries using neutral aqueous solution as electrolyte.

Entry	Anode-active Material	Cathode-active Material	Electrolyte	Atmo-sphere	Capacity loss* (Rate)	Ref.
This Work	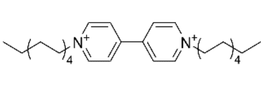	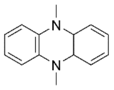	**3 M KCl aqueous solution**	**Open-air**	**−4.5% (15C)**	-
1	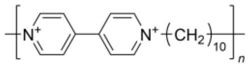	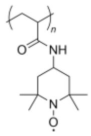	0.1 M $NaBF_4$ aqueous solution	Ar	−3%† (60C)	[6]
2	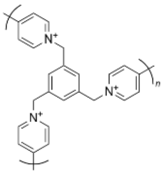	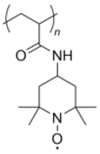	0.1 M NaCl aqueous solution	Ar	−5%†‡ (60 C)	[7]
3	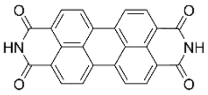	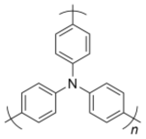	21 m KFSI aqueous solution	N/A§	−12%† (4.5 C)	[8]
4	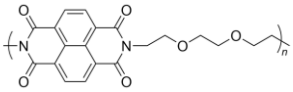	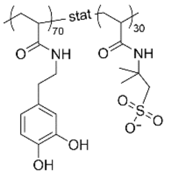	2.5 M $LiNO_3$ aqueous solution	Ar	−5%† (30 C)	[9]
5	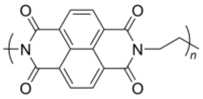	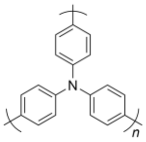	21 m LiTFSI aqueous solution	N/A§	−12%† (4.6 C)	[10]
6	Anthraquinone crosslinked polymer	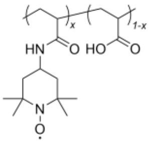	3 M NaCl aqueous solution	Sealed Cell	−19%† (10 C)	[11]
7	Anthraquinone crosslinked polymer	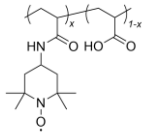	3 M NaCl aqueous solution	N_2	−3%† (5 C)	[12]

* Discharge capacity loss from the highest capacity after 100 cycles unless otherwise stated.

† Value estimated from the charge–discharge graph in the relevant reference.

‡ Capacity loss after 200 cycles.

§ Not stated in the relevant reference.

Table S2. Synthesis of viologen derivatives.

Entry	Structure	Molecular Weight	Water solubility	Ref.
1	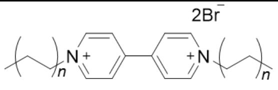 n = 3	514.38	Soluble	[1]
2	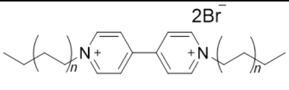 n = 4	598.54	Insoluble	[2]
3	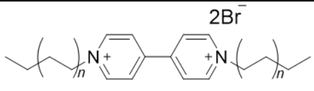 n = 5	654.65	Insoluble	[2]

References

[1] H.-C. Lu, S.-Y. Kao, H.-F. Yu, T.-H. Chang, C.-W. Kung, K.-C. Ho, *ACS Applied Materials & Interfaces* **2016**, *8*, 30351-30361.

[2] M. C. Grenier, R. W. Davis, K. L. Wilson-Henjum, J. E. LaDow, J. W. Black, K. L. Caran, K. Seifert, K. P. C. Minbiole, *Bioorganic & Medicinal Chemistry Letters* **2012**, *22*, 4055-4058.

[3] aK. Oka, S. Murao, M. Kataoka, H. Nishide, K. Oyaizu, *Macromolecules* **2021**, *54*, 4854-4859; bK. Oka, C. Strietzel, R. Emanuelsson, H. Nishide, K. Oyaizu, M. Strømme, M. Sjödin, *ChemSusChem* **2020**, *13*, 2280-2285; cK. Oka, S. Furukawa, S. Murao, T. Oka, H. Nishide, K. Oyaizu, *Chemical Communications* **2020**, *56*, 4055-4058.

[4] aC. DeBruler, B. Hu, J. Moss, X. Liu, J. Luo, Y. Sun, T. L. Liu, *Chem* **2017**, *3*, 961-978; bT. Hagemann, J. Winsberg, M. Grube, I. Nischang, T. Janoschka, N. Martin, M. D. Hager, U. S. Schubert, *Journal of Power Sources* **2018**, *378*, 546-554; cG. Kwon, S. Lee, J. Hwang, H.-S. Shim, B. Lee, M. H. Lee, Y. Ko, S.-K. Jung, K. Ku, J. Hong, K. Kang, *Joule* **2018**, *2*, 1771-1782; dI. Landa-Medrano, I. Lozano, N. Ortiz-Vitoriano, I. Ruiz de Larramendi, T. Rojo, *Journal of Materials Chemistry A* **2019**, *7*, 8746-8764; eM. Lee, J. Hong, B. Lee, K. Ku, S. Lee, C. B. Park, K. Kang, *Green Chemistry* **2017**, *19*, 2980-2985.

[5] K. Oka, H. Nishide, *Redox Polymers for Energy and Nanomedicine* **2020**, *34*, 137.

[6] K. Koshika, N. Chikushi, N. Sano, K. Oyaizu, H. Nishide, *Green Chemistry* **2010**, *12*.

[7] N. Sano, W. Tomita, S. Hara, C. M. Min, J. S. Lee, K. Oyaizu, H. Nishide, *ACS Appl Mater Interfaces* **2013**, *5*, 1355-1361.

[8] J. Ge, X. Yi, L. Fan, B. Lu, *Journal of Energy Chemistry* **2021**, *57*, 28-33.

[9] N. Patil, A. Mavrandonakis, C. Jérôme, C. Detrembleur, N. Casado, D. Mecerreyes, J. Palma, R. Marcilla, *Journal of Materials Chemistry A* **2021**, *9*, 505-514.

[10] X. Dong, H. Yu, Y. Ma, J. L. Bao, D. G. Truhlar, Y. Wang, Y. Xia, *Chemistry–A European Journal* **2017**, *23*, 2560-2565.

[11] K. Sato, R. Katagiri, N. Chikushi, S. Lee, K. Oyaizu, J.-S. Lee, H. Nishide, *Chemistry Letters* **2017**, *46*, 693-694.

[12] K. Hatakeyama-Sato, H. Wakamatsu, R. Katagiri, K. Oyaizu, H. Nishide, *Adv Mater* **2018**, *30*, e1800900.
